# Genome-Wide Association Study of Phenolic Content and Antioxidant Properties in Eggplant Germplasm

**DOI:** 10.3390/genes14071315

**Published:** 2023-06-22

**Authors:** Nayoung Ro, Mesfin Haile, Ho-Cheol Ko, Gyu-Taek Cho, Jungro Lee, Bichsaem Kim, Sookyeong Lee, Seong-Hoon Kim

**Affiliations:** National Agrobiodiversity Center, National Institute of Agricultural Sciences, Rural Development Administration, Jeonju 54874, Republic of Korea; mesfinhaile97@gmail.com (M.H.); hchko@korea.kr (H.-C.K.); gtcho@korea.kr (G.-T.C.); jrlee@korea.kr (J.L.); bsam92@korea.kr (B.K.); xsanta7@korea.kr (S.L.); shkim0819@korea.kr (S.-H.K.)

**Keywords:** antioxidant activity, eggplant, GWAS, SNPs, total phenolic content

## Abstract

The phenolic compounds in eggplant offer potential natural antioxidants for improved health. A large number of samples were examined in order to find eggplant germplasm with a high potential for health promotion. A genome-wide association study (GWAS) was conducted to identify single nucleotide polymorphisms (SNPs) associated with variations in total phenolic content (TPC) and antioxidant activity in eggplants, including ABTS (2,2′-azino-bis(3-ethylbenzothiazoline-6-sulfonic acid)) scavenging activity and ferric reducing antioxidant power (FRAP). TPC values varied from 14.19 to 842.90 mg gallic acid equivalent (GAE)/100 g of dry weight of eggplant fruit powder. TPC showed a strong positive correlation with both FRAP and ABTS (r = 0.89 *** and 0.77 ***, respectively). The GWAS identified 20 SNPs that were significantly associated out of 29,183 SNPs. Out of the 20 significant SNPs, 11 showed associations with TPC, 4 with ABTS activity, and 5 with FRAP. Among the SNPs associated with TPC, one SNP was found on each of Chromosomes 3, 4, 7, and 12. In contrast, Chromosome 5 comprised two SNPs associated to TPC. Furthermore, the gene encoding IRX12 laccase-4 on Chromosome 10 was found to contain five SNPs associated with TPC. Four significantly linked SNPs on Chromosomes 1 (1 SNP), 4 (2 SNPs), and 10 (1 SNP) were found to be related to ABTS activity. The identified SNPs will be further examined as markers for selecting desirable eggplant varieties and exploring the links between candidate genes, phenolic content, and antioxidant activity. The findings of this study could assist in further study and the development of eggplants with improved health advantages through targeted breeding.

## 1. Introduction

Eggplant (*Solanum melongena* L.), commonly known as aubergine, brinjal, berenjena, or guinea squash, is a valuable non-tuberous nightshade crop. Eggplants have been grown for centuries in Asia, Africa, Europe, and the Middle East [1]. According to FAO [2], China is the largest producer of eggplant (22.17 million tons), followed by India (9.89 million tons), Egypt (1.01 million tons), Turkey (0.86 million tons), and Iran (0.55 million tons). In 2021, the global production of eggplant was estimated to be 58.6 million tons [2].

Eggplant is a high-yielding and inexpensive agricultural crop that is grown in a wide variety of shapes, sizes, and colors. Because of its high nutritional value and extensive applications in the formulation of various types of fresh, canned, and frozen foods, such as pickled, grilled, fried, or stuffed eggplant, as well as different cuisines such as eggplant kibbeh, kashke bademjan, and several eggplant stews, global interest in eggplant farming is rapidly increasing [3,4]. The eggplant fruit not only contains proteins, dietary fiber, minerals, and minerals of interest such as potassium, calcium, magnesium, sodium, and iron [5], but it is also high in polyphenols, including phenolic acids such as chlorogenic acid, p-coumaric acid, and caffeic acid [6,7]. It also comprises flavonoids, including trace quantities of flavonols and a high content of various acylated and nonacylated anthocyanins, especially in purple-colored varieties of eggplant [8].

Among the cultivated members of the *Solanaceae* family, eggplant is regarded as the best source of total phenolic acids [9]. Phenolic compounds found in eggplant fruit have the ability to reduce abdominal glucose absorption while also providing cellular antioxidant defense, thereby avoiding oxidation and diabetes issues [10]. Hydroxycinnamic acid derivatives (mainly inflicted with amides or quinic acids) and anthocyanins (mainly delphinidin derivatives) were the most abundant phenolic compounds identified in eggplant [3,11]. Eggplant has been suggested to have plenty of health benefits, playing a significant role in the prevention of chronic diseases [3]. Several beneficial effects on human health have been ascribed to eggplant phenolic compounds, including antioxidant, anticarcinogenic, antidiabetic, anti-inflammatory, and cardioprotective activities [3].

Many studies have proven that eggplant has a wide range of phenolic content with prominent variability among eggplant lines [6,12,13,14]. The highest total phenolic content (TPC) was found in wild relatives of eggplant, including *Solanum incanum* L. and landraces [9,14]. Thus, landrace is another source of phenolic variation that can contribute to the selection of a successful breeding program. The reliance of agricultural genetic resources on reliable and readily available phenotypic data is a significant challenge in crop genetic resource selection [15]. The rapid advancements of genomics have provided crop breeders with the ability to develop stress-tolerant, disease-resistant, and high-yielding plants. Despite the difficulties, some research has been conducted to determine how the genetics of a plant affect phenolic compounds, antioxidant activities, carotenoids, and anthocyanin in different crops such as barley [16] and ornamental plants, particularly rose petals [17].

The genome-wide association study (GWAS) is a potent method that utilizes natural genetic variability to decode the genetic basis of complex phenotypes [18]. GWAS provides better mapping resolution and enables the detection of associations between molecular markers and desired traits. Therefore, it is an effective tool for identifying markers linked to desirable traits in various crops [19,20,21]. Its wide-ranging applications in various fields demonstrate its importance in advancing scientific understanding and improving human and crop health. In order to develop superior eggplant varieties with enhanced nutritional properties, it is essential to evaluate a large pool of eggplant germplasm for key chemical components and properties, such as TPC and antioxidant activity. However, analyzing such a large number of genetic resources can be a daunting task. In order to quickly and effectively choose the most promising germplasm for further breeding, genetic association studies can be a useful tool in discovering specific genetic markers linked to significant chemical properties.

This study aims to leverage the genetic diversity of eggplant germplasm and identify the possible underlying genetic factors that contribute to the TPC and antioxidant activity (ABTS and FRAP) of eggplants. TPC and antioxidant activity are crucial components that provide health benefits to consumers, making them ideal candidates for selection and improvement. Through a comprehensive analysis of the genetic factors linked to these beneficial traits, the understanding of underlying mechanisms and identification of potential genetic markers will assist future breeding programs in developing eggplant varieties with enhanced nutritional value.

## 2. Materials and Methods

### 2.1. Plant Materials 

A total of 224 eggplant accessions collected from different countries across the world, including germplasm from Korea, were among the established core collection. These germplasms belong to different species. The eggplant seedlings (eight to ten in triplicate) were planted in the National Agrobiodiversity Center (NAC) greenhouse at the Rural Development Administration (RDA), Jeonju, the Republic of Korea. The eggplants were cultivated according to the RDA-recommended eggplant cultivation method. The introduction number (IT), species name, and geographic origin of the 224 eggplant germplasms are presented in Supplementary Table S1.

### 2.2. Sample Preparation, Extraction and Analysis

The extraction of phenolic compounds was carried out with 70% acetone as a solvent. Briefly, 1.0 g of eggplant powder was mixed with 15 mL of the solvent in a 45 mL extraction tube. The mixture was sonicated for 25 min at 25 degrees Celsius in the dark, and the supernatant was collected via centrifugation at 4000 rpm for 10 min. Total phenolic content (TPC), ABTS+ scavenging activity, and ferric reducing antioxidant power (FRAP) assays were determined using these extractions.

### 2.3. Total Phenolic Content 

The Folin–Ciocalteu method [22] was used to determine the TPC of each eggplant sample with minor modifications. The phenolic extract (100 µL) was mixed with an equal volume of Folin–Ciocalteu reagent in the dark at 25 °C After 3 min, 100 µL of a 2% Na_2_CO_3_ solution was added to the mixture and incubated for an additional 30 min in the dark. The absorbance was then measured at 750 nm (Eon Microplate Spectrophotometer, Bio-Tek, Winooski, VT, USA), and TPC was calculated as gallic acid equivalent (mg) per gram of eggplant powder (mg GAE/g), based on triplicate measurements. 

### 2.4. ABTS Radical Scavenging Activity

ABTS assay was conducted according to the method described by Re et al. [23] with minor modifications. A mixture of 7.0 mM ABTS and 2.45 mM potassium persulphate was prepared and stored in dark conditions for 16 h. A 190 μL working solution of ABTS^+^ was combined with 10 μL of sample extract and the mixture was incubated at 25 °C in the dark. After 3 min, the absorbance at 734 nm was determined (Eon Microplate Spectrophotometer, Bio-Tek, Winooski, VT, USA). The activity of ABTS^+^ scavenging was evaluated in triplicate and expressed in milligrams of ascorbic acid equivalent per gram of eggplant powder weight (mg AAE/100 g). 

### 2.5. Ferric Reducing Antioxidant Power (FRAP) Assay

FRAP analysis was conducted according to the method described by Yen and Chen [24] and subsequently modified [25]. A 1.5 mL reaction tube was filled with 60 µL of the sample extract. Subsequently, 150 µL of freshly prepared phosphate buffer (pH 6.6, 0.2 M) and an equal volume of 1% potassium ferricyanide (K3Fe(CN)6) were added. After incubating the mixture at 50 °C for 20 min, 150 µL of 10% trichloroacetic acid was added. The resulting mixture was then centrifuged at 3000 rpm for 10 min. Following centrifugation, 20 µL of a 0.1% ferric chloride solution was added to 100 µL of distilled water and 100 µL of the upper supernatant. The solution was incubated for an additional 10 min and tested for absorbance at 700 nm using an Eon Microplate Spectrophotometer (Bio-Tek, Winooski, VT, USA). The FRAP activity was expressed as ascorbic acid equivalent (mg) per gram of dried fruit powder weight (mg AAE/g).

### 2.6. DNA Extraction and Genotyping by Sequencing (GBS)

The Genomic DNA Prep Kit (Inclone Biotech, Gyeonggi-do, Republic of Korea) was used to extract DNA from all 224 eggplant samples, following the manufacturer’s protocol. The Illumina HiSeq X Ten sequencing platform was used for sequencing, using paired-end reads with an average length of 151 bp. The detailed GBS statistics for 224 eggplant accessions are presented in Supplementary Table S2. The restriction enzyme ApeKI (5’-GCWGC-3’) was used to construct GBS libraries according to a modified protocol [26]. The oligonucleotides containing the top and bottom strands of each barcode adapter and a common adapter were diluted separately with TE at a concentration of 50 µM, and then annealed with a thermocycler. Adapter-containing wells were loaded with DNA samples (100 ng/L). Sample digestion (DNA with adapters) was performed overnight at 75 °C with ApeKI (New England Biolabs, Ipswich, MA, USA). The DNA samples, each containing a unique barcode adapter, were pooled (5 L) and purified using a commercial kit (QIAquick PCR Purification Kit; Qiagen, Valencia, CA, USA) according to the manufacturer’s instructions. Each library’s restriction fragments were amplified in 50 µL volumes containing 2 µL of pooled DNA fragments, HerculaseII Fusion DNA Polymerase (Agilent, CA, USA), and 25 pmol of forward and reverse primers: (A) 5’-AATGATACGGCGACCACCGAGATCTACACTCTTTCCCTACACGACGCTCTTCCGATCT-3’ and (B)5’-CAAGCAGAAGACGGCATACGAGATCGGTCTCGGCATTCCTGCTGAACCGCTCTTCCGATCT-3’.

Raw sequences were demultiplexed into 224 samples in line with the barcode sequences. Adapter and barcode sequences were eliminated using the software Cutadapt (version 1.8.3) [27]. DynamicTrim and LengthSort programs of the SolexaQA (v.1.13) package [28] were used to remove low-quality sequences. A Phred score ≥20 was used as the criterion for DynamicTrim, and a read length of ≥25 pb was applied for LengthSort. BWA (Burrows–Wheeler Aligner, ver.0.6.1-r104) [29] generated clean reads, passed the preprocessing process, and performed mapping to the reference genome of *Solanum melongena* L. (https://solgenomics.net/ accessed on 19 September 2022). Mapping was a preliminary step to detect raw SNPs (In/Del) between the *S. melongena* genome (Eggplant Genome Consortium V4.1) and sequenced samples.

### 2.7. SNP Calling and Filtering 

Clean reads were mapped to the reference genome sequence, and the obtained SAM files were used to discover raw SNPs using SAMtools (0.1.16) [30] and extract consensus sequences. SNP validation was conducted using SEEDERS in-house script [31] before SNP detection; raw SNP detection was performed, and default values were used except for the following options: a minimum mapping quality for SNPs (−Q) of 30, minimum mapping quality for gaps (−q) of 15, minimum read depth (−d) of 3, minimum InDel score for nearby SNP filtering (−G) of 30, SNPs within INT bp around a gap to be filtered (−w) of 15, window size for filtering dense SNPs (−W) of 30, and maximum read depth (−D) of 165. An integrated SNP matrix was obtained between samples to assess SNPs between the assessed objects. A list of unions was generated by comparing each sample’s raw SNP sites to a standard template, and a non-SNP locus was filled in from the sample’s consensus sequence. The final SNP matrix was formed by filtering out the miscalled SNP sites using SNP comparison among samples. Based on the position, SNPs were classified as homozygous (SNP read depth ≥ 90%), heterozygous (40% ≤ SNP read depth ≤ 60%), etc. (homozygous/heterozygous; could not be separated by type). Based on the location information of the reference genome sequence (*Solanum melongena* L), the designated SNP positions were defined as intergenic or genic regions. 

### 2.8. Population Structure and Genome-Wide Association Analysis

Structure software [32,33] was used to conduct the population structure analysis. Among 29,183 filtered SNPs of 224 eggplant germplasms, randomly selected SNPs (14,592) were used for population structure analysis. A Bayesian model-based strategy was implemented, with 10,000 burns in the period and 10,000 Markov chain Monte Carlo (MCMC) steps. K values were set from 1 to 10, and the number of iterations was set to 10 to find the proper K (population). The appropriate number of populations (K value) was estimated based on the delta K (ΔK) method [34] using web-based STRUCTURE HARVESTER [35]. 

Association analysis was conducted using 29,183 SNP datasets using a linear mixed model (LMM) [36]. QTLmax 3.0 [37] genetic analysis software was used to conduct the association analysis. Minor alleles demonstrating allelic frequencies of less than 5% were excluded from the analysis. The threshold for describing a marker as significant was taken at −log 10 (*p* < 0.0001) or more [38]. The Basic Local Alignment Search Tool (BLAST) was used to find the adjacent genes where SNPs were found in the eggplant genome database (eggplant genome consortium V4.1, https://solgenomics.net (accessed on 19 September 2022)).

### 2.9. Statistical Analysis 

The Microsoft Excel program was used for data summarization and descriptive statistics on TPC, ABTS, and FRAP. Principal component analysis and correlation were computed using R software (version 4.2.1). Other statistical programs and packages are mentioned in the respective sections where they were employed.

## 3. Results

### 3.1. Eggplant Germplasm Variation in TPC, ABTS and FRAP

**Figure 1:**
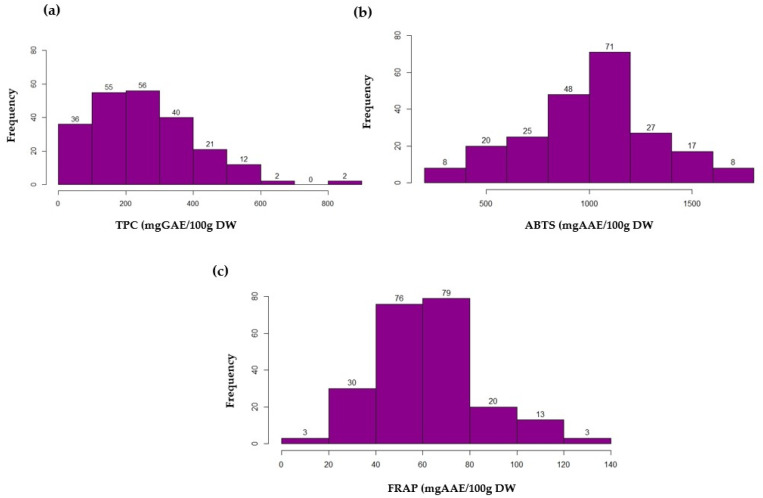
The frequency distribution of 224 eggplant germplasms based on the TPC and antioxidant activity (ABTS and FRAP). (**a**) Frequency distribution based on TPC. (**b**) Frequency distribution based on ABTS. (**c**) Frequency distribution based on FRAP. The x-axis shows the TPC, ABTS, and FRAP values while the y-axis shows the number of eggplant germplasms.

In this study, 224 eggplant accessions from different countries and species were tested for their phenolic content and antioxidant activity using the ABTS and FRAP assays. There was a large variation in the TPC and antioxidant properties of the eggplant germplasm collected and deposited at the RDA gene bank. The TPC ranged from 14.19 to 844.57 mg gallic acid equivalent (GAE)/100 g of dried fruit powder (Table 1). The ABTS activity was expressed in the amount of ascorbic acid equivalent and ranged from 259.87 to 1727.27 mg ascorbic acid equivalent (AAE)/100 g DW. The FRAP activity was estimated in terms of the ascorbic acid equivalent and ranged from 3.80 to 133.25 mg AAE/100 g DW. Two *S. melongena* germplasm collections, K145198 (ID: 557) and K168113 (ID: 607), had the highest TPC, ABTS, and FRAP of all the tested germplasm. The number of germplasm distributions based on TPC, ABTS, and FRAP is depicted in Figure 1. A large number of germplasms (112) had a TPC within the range of 100–300 mg GAE/100 g DW (Figure 1a). On the other hand, four accessions had between 600 and 845 mg GAE/100 g DW. Regarding ABTS, about eight germplasms had greater than 1500 mg AAE/100 g DW (Figure 1b). In terms of FRAP, a large number of germplasms (155 germplasms) had between 40 and 80 mg AAE/100 g DW (Figure 1c).

### 3.2. Correlation and Principal Component Analysis (PCA)

**Figure 2:**
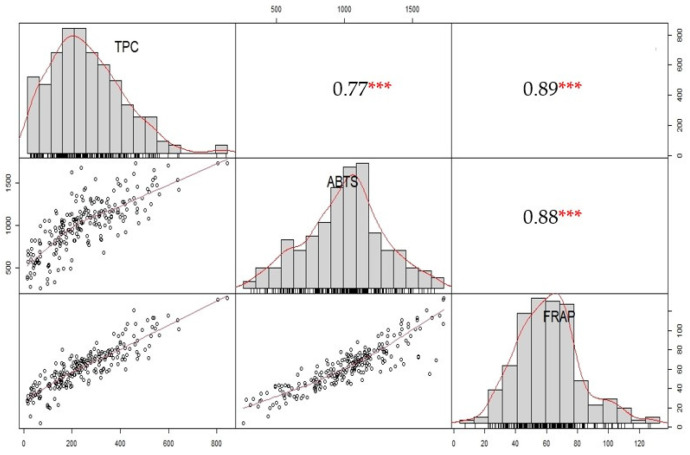
Correlation analysis of TPC, ABTS, and FRAP using 224 eggplant genetic resources. Each box represents a Pearson correlation value (*** indicates significance level *p* < 0.001).

The correlation among chemical traits was examined using 224 eggplant accessions. The results, presented in Figure 2, revealed a significant positive correlation between TPC and the ABTS activity (r = 0.77 ***), as well as between TPC and FRAP (r = 0.89 ***). Furthermore, a strong positive correlation (r = 0.88 ***) was observed between ABTS and FRAP. These findings indicate that the increased antioxidant activity, as measured by both the ABTS and FRAP assays, may be associated with the higher TPC. These correlations provide valuable insights into the relationship among the studied traits of eggplant accessions.

**Figure 3:**
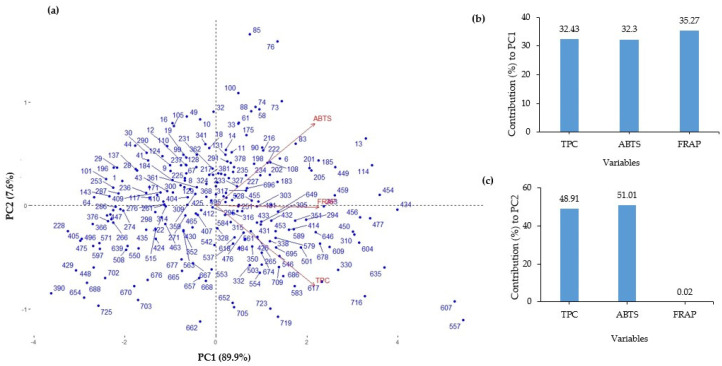
Principal component analysis of TPC, ABTS, and FRAP values from 224 eggplant accessions. (**a**) PCA variables and individual observations. (**b**) Contribution of variables to PC1. (**c**) Contribution of variables to PC2.

PCA was conducted to explore the relationships among the chemical traits (TPC, ABTS, and FRAP) in 224 eggplant accessions (Figure 3a). The analysis revealed three principal components (PC1, PC2, and PC3), which collectively accounted for 99.00% of the total variance. PC1 explained the highest variance (89.90%), followed by PC2 (7.60%) and PC3 (2.50%). The contribution of variables to each principal component was examined. In PC1, TPC, ABTS, and FRAP showed respective contributions of 32.43%, 32.3%, and 35.27% (Figure 3b). This suggests that these three variables collectively explained a substantial portion of the variance captured by PC1. In PC2, TPC and ABTS demonstrated significant contributions of 48.91% and 51.01%, respectively, while FRAP exhibited a negligible contribution of 0.02% (Figure 3c). These findings indicate that TPC and ABTS primarily account for the variance observed in PC2, while FRAP has limited influence. Accessions with higher values for all three variables tend to cluster together in the same direction on the PCA plot, reflecting their overall similarity in terms of these three variables. For example, the following accessions are the top five in terms of their respective chemical content: accession IDs 557, 607, 716, 635, and 434 are higher in TPC content; accession IDs 557, 607, 13, 434, and 76 are higher in ABTS; and accession IDs 557, 607, 114, 434, and 477 are higher in FRAP. The accession IDs and their standard numbers (introduction numbers) can be found in Supplementary Table S1. 

### 3.3. Population Structure Analysis 

**Figure 4:**
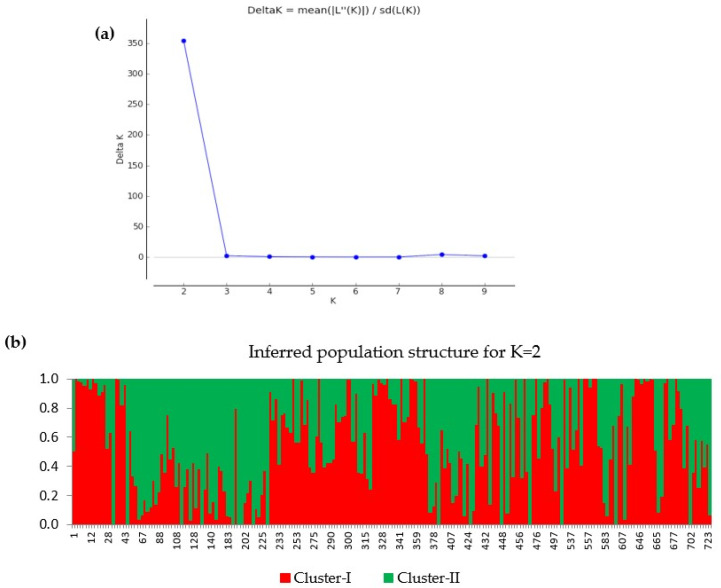
Population structure analysis results for 224 eggplant accessions. (**a**) Prediction of the number of groups (K = 2) based on the Evanno method. (**b**) The relatedness of eggplant accession based on structure analysis indicates that there are two groups within the eggplant population. Each cluster is represented by a different color: red (Cluster-I) and green (Cluster-II).

The results obtained from the population structure analysis conducted on 224 eggplant accessions are presented in Figure 4**.** The Evanno method was used to predict the number of groups (K = 2) in the population. As depicted in Figure 4a, the method suggests that the eggplant population can be divided into two distinct groups. Further insights can be gained from Figure 4b, which illustrates the relatedness among the eggplant accessions based on the population structure analysis. The figure reveals the presence of two clusters, represented by different colors: red (Cluster-I) and green (Cluster-II). According to the results, Cluster-I consisted of 124 accessions, while Cluster-II comprised 100 accessions (Supplementary Table S3). This indicates the presence of two genetically distinct subgroups within the analyzed eggplant population.

### 3.4. Genome-Wide Association Analysis

**Figure 5:**
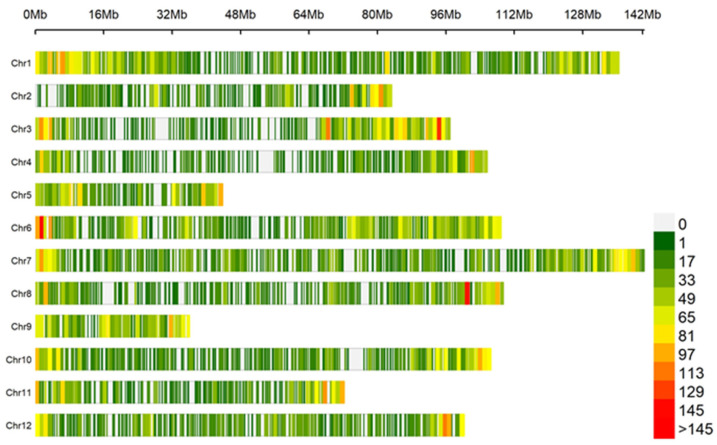
The distribution of SNPs across all 12 chromosomes from 224 eggplant accessions, using a 1 Mb window size. The density of the SNPs is represented by heatmap colors, providing a visual representation of their distribution patterns.

**Figure 6:**
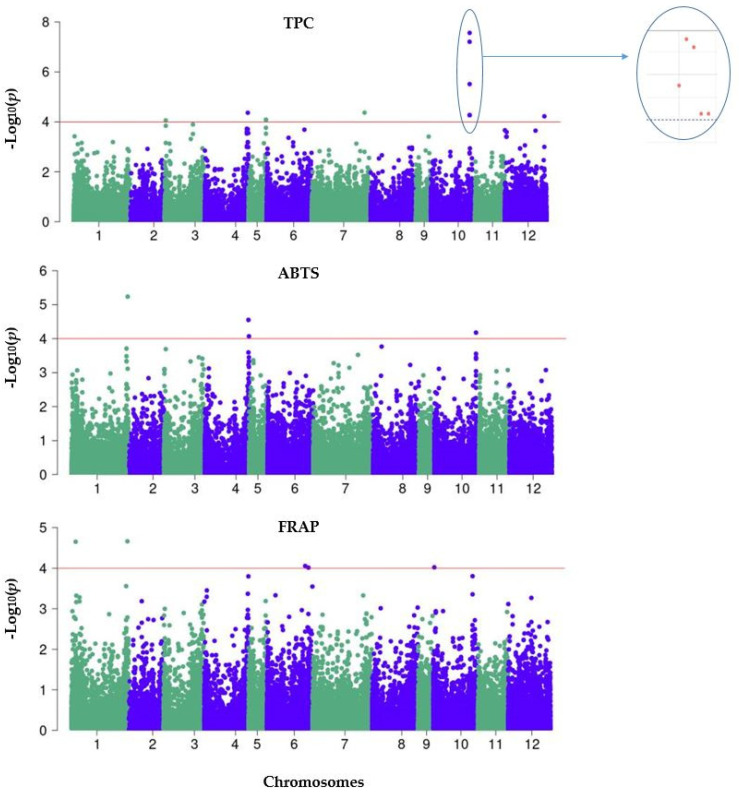
Manhattan plots depicting the association of TPC, ABTS, and FRAP using 224 eggplant genetic resources. Each dot represents a single SNP, with the x-axis showing genomic location (chromosomes: colored and labeled) and the y-axis showing association level (−log_10_ (*p*) = 4.0). The horizontal red line represents the significance threshold for the association of SNPs with traits.

**Figure 7:**
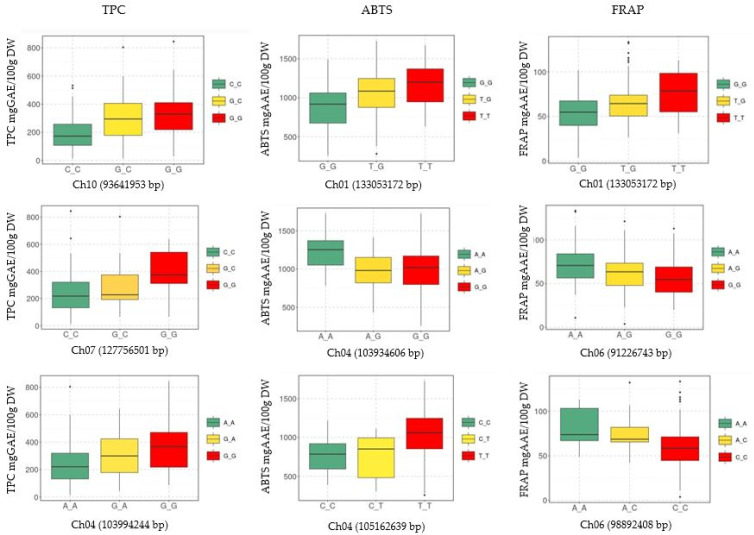
Box plots showing the allelic effect of selected significantly associated SNP markers for TPC, ABTS, and FRAP values. The y-axis represents the average TPC, ABTS, and FRAP values for each genotype, which are represented by different alleles. The x-axis represents alleles found at specific chromosomes and positions.

Association analysis was conducted using 29,183 SNPs generated from 224 eggplant genetic resources. The distribution of SNPs within a 1 Mb window size across all 12 chromosomes can be seen in Figure 5. The study identified a total of 20 SNPs significantly associated with TPC, ABTS, and FRAP. Among these, 11 SNPs were associated with TPC, 4 with ABTS, and 5 with FRAP. The results of the association analysis are presented in the Manhattan (Figure 6) and quantile–quantile (Q–Q) plots (Supplementary Figure S1). Additionally, box plots were used to compare and visualize the average trait values by examining the allelic frequency at specific markers within the GWAS panels, aiming to investigate the relationship between genetic variations and trait expression. Figure 7 displays box plots demonstrating the allelic effects of selected SNP markers significantly associated with TPC, ABTS, and FRAP.

Regarding the ten SNPs associated with TPC, one SNP was found on each of Ch03, Ch04, Ch07, and Ch12, while two SNPs were discovered on Ch05. Five SNPs were identified on Ch10, located at 93641953 bp (C/G), 93641970 bp (T/A), 93641946 bp (A/G), 93642015 bp (C/T), and 93642000 bp (A/T), within the gene that encodes IRX12 laccase-4. The two SNPs (42339531 bp: T/G and 42339551 bp: G/T) associated with TPC on Ch05 were found in genes encoding PSL4 glucosidase 2 subunit beta. In Figure 7, SNP markers located at 93641953 bp (Ch10), 127756501 bp (Ch07), and 103994244 bp (Ch04) revealed that individuals with GG alleles had relatively higher average TPC content compared to other alleles at those specific marker positions.

Four SNPs were associated with ABTS activity in eggplant accessions. Two of these SNPs were located in the intergenic regions on Ch01 and Ch04, while the other two were found within genes encoding glyceraldehyde-3-phosphate dehydrogenase (GAPB) on Ch04 (105162639 bp) and putative disease resistance protein (RGA4) on Ch10 (99069998 bp) (Table 2). Based on the SNP marker at position 133053172 bp (Figure 7), individuals with TT alleles had higher average ABTS activity compared to those with TG and GG alleles. This marker was located in the intergenic region on Ch01.

Furthermore, a total of five SNPs were significantly associated with FRAP. Among them, two SNPs were found on Ch01, one in the intergenic region (133053172 bp) and the other within a gene encoding a serine/threonine-protein kinase. On Chromosome 6, the remaining two SNPs were located in the intergenic region at 91226743 bp and within a gene of unknown function at 98892408 bp. The average FRAP values of genotypes with AA alleles at these specific marker positions on Ch06 were higher compared to individuals with other alleles, as shown in Figure 7.

## 4. Discussion

The variations in phenolic content and antioxidant activity were evaluated in 224 eggplant genetic resources. Additionally, a genome-wide association analysis was conducted to identify SNPs that exhibited significant associations. The TPC and antioxidant activity varied greatly. The TPC ranged from 14.19 to 844.57 mg GAE/100 g. Reports have also shown a wide range in TPC (range: 22–20,490 mg/kg fresh weight) contents in the peel of eggplants [11,39,40,41]. These variations could be attributed to a variety of factors, including ecological differences and genetic makeup differences. Previous research has shown that genotype [42], crop management approaches [43], post-harvest storage temperature, and processing extent all had a significant impact on antioxidant levels in eggplant [44,45]. The principal phenolic compounds in eggplant fruits have been shown to be particularly advantageous for human health owing to their proven biological actions, and they may be utilized to treat a variety of metabolic and cardiovascular illnesses [46]. The study revealed a strong positive correlation between TPC and antioxidant activity, in line with several findings [47,48,49]. This association may be attributed to the fact that phenolic acids and flavonoids, which are predominantly found in TPC, contribute significantly to the antioxidant activity of plants [48]. The germplasm collections of *S. melongena*, namely K145198 (ID: 557) and K168113 (ID: 607), exhibited higher levels of TPC, ABTS, and FRAP compared to other tested germplasm collections. Furthermore, the PCA analysis demonstrated that these two germplasm collections were distinct and positioned far away from other accessions (Figure 3a). These two accessions were also grouped in Cluster-II, as shown in the population structure analysis (Figure 4 and Supplementary Table S3).

Molecular breeding is a powerful tool for crop improvement and has been used successfully in a variety of crop species. GWAS enables the mapping of genetic regions associated with economically relevant traits in crop species, including yield, resistance to biotic and abiotic stress, and quality [50,51,52,53,54]. This information has also been used in breeding programs to undertake marker-assisted selection (MAS) to find genes underlying phenotypic variation [55]. We identified 20 SNPs (*p* < 0.0001) associated with phenolic compounds and antioxidant activity in eggplant, highlighting the genetic regulation of these traits in the crop. These SNPs have the potential to serve as genetic markers for breeding programs aimed at enhancing phenolic content and antioxidant activity. Similarly, a study on Tibetan wild barley revealed the identification of 20 unique QTLs (*p* < 0.001) associated with phenolic compounds, flavonoid content, and antioxidant activity [16]. The researchers also suggested that the divergence of these QTLs between wild and cultivated barley populations indicates the impact of domestication on genetic diversity and the potential loss of specific genes or alleles controlling phenolic traits in cultivated barley [16]. Even though phenolic acids are constituents of phenolic compounds and could potentially contribute to TPC and antioxidant activity, no common SNPs were found to be associated with both TPC and antioxidant activity in this study. However, a previous study identified a shared locus (bPb-0836) that was associated with both TPC and antioxidant activity [16]. The lack of common SNPs associated with both TPC and antioxidant activity in the current study compared to a previous study could be attributed to genetic variability, differences in experimental design, marker coverage, statistical power, and the complex nature of the traits. 

On Chromosome 10, five SNPs significantly associated with TPC were found in a gene that encodes IRX12 laccase-4 (Table 2). Several research findings indicated that laccases (LACs) are involved in the biosynthesis of lignin, which is known to be made up of phenolic compounds. Plant laccase (LAC) enzymes, which are members of the blue copper oxidase/p-diphenol:dioxygen oxidoreductase family, have been implicated in lignin production; these enzymes polymerize monolignols into lignin [56,57,58]. Laccases have been cloned and characterized in gymnosperms, monocots, and eudicots, indicating that they played a crucial role in the evolution of the plant vascular system [57,58,59,60,61]. Among the 17 known members of the laccase family in Arabidopsis [60,61], 4 (LAC4, LAC11, LAC15, and LAC17) have been identified as being involved in lignin production [62,63,64]. Lignin is a heterogeneous, complex phenolic polymer that accumulates in the cell walls of particular cell types [65]. Lignin gives strength and stiffness to the secondary cell walls of tracheary components, sclereids, and fibers in vascular plants. Lignin accumulation occurs in three stages: biosynthesis of phenolic monomers, mostly phenylpropanoids, in the cytoplasm [66]; export into the apoplast [67]; and subsequent oxidative polymerization in the cell wall by radical coupling catalyzed by laccases (LACs) and class III peroxidases (PRXs) [68]. Among the phenoloxidases linked with lignin, *Arabidopsis* (*Arabidopsis thaliana*) and poplar (*Populus* sp.) specific paralogs of LACs are the key enzymes necessary to accumulate lignin in vascular tissues, although their influence on lignin chemistry is unknown [68]. LACs may represent the primary regulatory components capable of channeling extracellular phenolic compounds toward lignin because distinct LAC paralogs are unique to lignifying conditions and have been proposed to induce the transport of phenylpropanoids [67]. Additional investigation is required to determine any potential association of this gene to phenolic compounds.

Pleiotropy is a sign that related traits might possibly have some genetic factors in common [69]. One pleiotropic SNP (Ch01: 133053172) was found to have a significant association with both ABTS and FRAP. This SNP is located in the intergenic region. Among the SNPs associated with ABTS, the adjacent genes were a putative disease resistance protein (RGA4) and glyceraldehyde-3-phosphate dehydrogenase (GAPB). SNPs associated with FRAP were found in a gene encoding a serine/threonine-protein kinase (haspin homolog), aspartic protease in guard cell 1 (ASPG1) protein, and a protein with an unknown function. Additionally, four SNPs were found in the intergenic region for both ABTS and FRAP, with two SNPs in each. SNPs are commonly located in coding and noncoding regions, as well as intergenic regions of genomes. They have varying abundance across these genomic regions, with a frequency of approximately 1 SNP per every 100–300 base pairs of DNA [70]. Intergenic spacers are often the focus of genetic diversity characterization [71]. While polymorphisms in coding regions can impact gene function [72,73,74], the majority of functionally significant variants are found in intergenic regions. This trend holds true across various species. For instance, in maize, high-resolution GWASs targeting multiple traits have revealed that 70% of significant functional associations are located in intergenic regions [75]. Therefore, the assessment of SNPs identified in the intergenic regions as potential markers for the desired trait is highly valuable. Figure 7 illustrates the variation in mean values of TPC and antioxidant activity based on the frequency of different alleles at specific marker positions. The observed differences highlight the potential impact of these markers on the traits of interest. In addition to the markers presented in this study, further marker SNPs will be evaluated as potential indicators, both individually and in combination, for selecting eggplant accessions with high TPC and antioxidant activity. The development of markers can greatly assist selection and breeding programs aimed at developing nutritionally enriched eggplant varieties.

## 5. Conclusions

Molecular plant breeding tools and functional genomics approaches have the potential to accelerate the development of crops with enhanced nutritional and health-promoting properties, improving human health and well-being. The germplasm collections of *S. melongena* with the codes K145198 and K168113 have higher levels of TPC, ABTS, and FRAP compared to other tested germplasm collections. These collections could be useful for future breeding and research programs aimed at developing eggplant varieties with improved nutritional and antioxidant properties. The SNPs identified in this study that were associated with TPC, ABTS, and FRAP, as well as the adjacent genes where the SNPs were identified, can help us understand the genetic associations and assist in developing effective markers for breeding programs. The present study identified SNPs associated with TPC in genes that encode IRX12 laccase-4, a protein critical to lignin biosynthesis, which includes the biosynthesis of phenolic monomers, mostly phenylpropanoids. These SNPs may shed light for further study to understand the relationship between IRX12 laccase-4 and TPC in plants. Further investigation of these SNPs is necessary to understand their potential use as markers in future studies and gain a deeper understanding of the mechanisms involved in phenolic synthesis and antioxidant activity.

## Figures and Tables

**Table 1:** Summary of the descriptive statistics of TPC (mg GAE/100 g DW), ABTS (mg AAE/100 g DW), and FRAP (mg AAE/100 g DW) using 224 eggplant germplasms.

	TPC	Antioxidant Activity	
		ABTS	FRAP
Mean	251.90	1010.15	61.94
Standard error	10.22	20.79	1.48
Standard deviation	152.91	311.10	22.23
Minimum	14.19	259.87	3.80
Maximum	844.57	1727.27	133.25
Count	224	224	224

**Table 2:** SNPs that were significantly associated with TPC, ABTS, and FRAP.

Traits	Chr.	Ref. Allele	Alt. Allele	Pos.	−Log_10_(*p*)	Genic/Intergenic	Description	Minor Allele	Major Allele
TPC	10	C	G	93641953	7.56	SMEL_010g352340.1	IRX12 Laccase-4	G	C
	10	T	A	93641970	7.21	SMEL_010g352340.1	IRX12 Laccase-4	A	T
	10	A	G	93641946	5.51	SMEL_010g352340.1	IRX12 Laccase-4	G	A
	7	C	G	127756501	4.37	SMEL_007g287460.1	PES Pescadillo homolog	G	C
	4	A	G	103994244	4.37	SMEL_004g221900.1	Transcription factor RF2b	G	A
	10	C	T	93642015	4.27	SMEL_010g352340.1	IRX12 Laccase-4	T	C
	10	A	T	93642000	4.27	SMEL_010g352340.1	IRX12 Laccase-4	T	A
	12	C	T	95274028	4.22	SMEL_012g395920.1	Bifunctional aspartokinase/homoserine dehydrogenase, chloroplastic	T	C
	5	T	G	42339531	4.08	SMEL_005g240560.1	PSL4 Glucosidase 2 subunit beta	G	T
	5	G	T	42339551	4.08	SMEL_005g240560.1	PSL4 Glucosidase 2 subunit beta	T	G
	3	T	C	2830477	4.06	SMEL_003g171200.1	Tyrosyl-DNA phosphodiesterase 1 (TDP1)	C	T
ABTS	1	T	G	133053172	5.24	Intergenic	-	T	G
	4	G	A	103934606	4.55	Intergenic	-	A	G
	10	A	G	99069998	4.18	SMEL_010g355130.1	Putative disease resistance protein (RGA4)	G	A
	4	T	C	105162639	4.08	SMEL_004g223250.1	Glyceraldehyde-3-phosphate dehydrogenase (GAPB)	C	T
FRAP	1	T	G	133053172	4.66	Intergenic	-	T	G
	1	C	T	9596945	4.65	SMEL_001g124000.1	Serine/threonine-protein kinase haspin homolog	C	T
	6	A	G	91226743	4.06	Intergenic	-	A	G
	10	C	A	2463130	4.02	SMEL_010g337760.1	Aspartic protease in guard cell 1 (ASPG1)	A	C
	6	C	A	98892408	4.02	SMEL_006g260190.1	Protein of unknown function	A	C

## Data Availability

Additional datasets, apart from the Supplementary Materials, can be obtained upon request from the corresponding author. The SNP data generated from the 288 eggplant accessions, including the ones in our study, have been submitted to the National Agricultural Biotechnology Information Center (NABIC) and can be accessed using the accession number NV-0776.

## Supplementary Materials

The following supporting information can be downloaded at https://www.mdpi.com/article/10.3390/genes14071315/s1, Table S1: the information for 224 eggplant germplasms, Table S2: the GBS analysis statistics for 224 eggplant accessions, Table S3: population structure analysis results of 224 eggplant accessions, Figure S1: the quantile–quantile (Q–Q) plots for the total phenolic content (TPC), ABTS, and FRAP values in eggplant germplasm.
