# Supplementary material for: Genome-Wide Association Study of Phenolic Content and Antioxidant Properties in Eggplant Germplasm
Source: Genes (Basel). 2023 Jun 22;14(7):1315. doi: 10.3390/genes14071315 (PMC10379237; doi:10.3390/genes14071315)
Supplement: Supplementary file 1 [file genes-14-01315-s001.zip › Supplementary Figure S1.pdf]

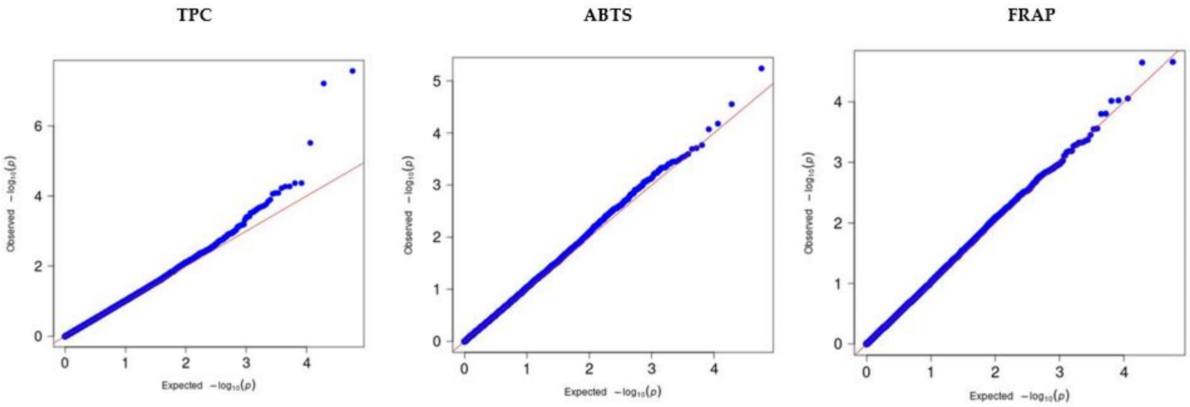

Supplementary Figure S1. The quantile-quantile (Q-Q) plots for the Total Phenolic Content (TPC), ABTS, and FRAP values in eggplant germplasm.
